# Triglyceride glucose index for predicting cardiovascular outcomes after percutaneous coronary intervention in patients with type 2 diabetes mellitus and acute coronary syndrome

**DOI:** 10.1186/s12933-020-01006-7

**Published:** 2020-03-10

**Authors:** Xiaoteng Ma, Lisha Dong, Qiaoyu Shao, Yujing Cheng, Sai Lv, Yan Sun, Hua Shen, Zhijian Wang, Yujie Zhou, Xiaoli Liu

**Affiliations:** Department of Cardiology, Beijing Anzhen Hospital, Capital Medical University, Beijing Institute of Heart Lung and Blood Vessel Disease, Beijing Key Laboratory of Precision Medicine of Coronary Atherosclerotic Disease, Clinical Center for Coronary Heart Disease, Beijing, 100029 China

**Keywords:** Triglyceride glucose index, Adverse cardiovascular outcomes, Type 2 diabetes mellitus, Acute coronary syndrome, Percutaneous coronary intervention

## Abstract

**Background:**

The triglyceride glucose (TyG) index, a simple surrogate estimate of insulin resistance, has been demonstrated to predict cardiovascular (CV) disease morbidity and mortality in the general population and many patient cohorts. However, to our knowledge, the prognostic usefulness of the TyG index after percutaneous coronary intervention (PCI) in patients with type 2 diabetes mellitus (T2DM) and acute coronary syndrome (ACS) has not been determined. This study aimed to evaluate the association of the TyG index with adverse CV outcomes in patients with T2DM and ACS who underwent PCI.

**Methods:**

The TyG index was calculated using the formula ln[fasting triglycerides (mg/dL) × fasting glucose (mg/dL)/2]. The primary endpoint was the composite of all-cause mortality, non-fatal stroke, non-fatal myocardial infarction, or unplanned repeat revascularization. The association between the TyG index and adverse CV outcomes was assessed by Cox proportional hazards regression analysis.

**Results:**

In total, 776 patients with T2DM and ACS who underwent PCI (mean age, 61 ± 10 years; men, 72.2%) were included in the final analysis. Over a median follow-up of 30 months, 188 patients (24.2%) had at least 1 primary endpoint event. The follow-up incidence of the primary endpoint rose with increasing TyG index tertiles. The multivariate Cox proportional hazards regression analysis adjusted for multiple confounders revealed a hazard ratio for the primary endpoint of 2.17 (95% CI 1.45–3.24; *P* for trend = 0.001) when the highest and lowest TyG index tertiles were compared.

**Conclusions:**

The TyG index was significantly and positively associated with adverse CV outcomes, suggesting that the TyG index may be a valuable predictor of adverse CV outcomes after PCI in patients with T2DM and ACS.

## Background

In the last few years, acute coronary syndrome (ACS), the most severe ischaemic heart disease, has been one of the leading causes of death worldwide [1]. Some patients with ACS remain at high risk for recurrent cardiovascular (CV) events despite the use of current guideline-recommended therapies, including prompt coronary revascularization, dual anti-platelet therapy, and intensive lipid-lowering therapy. This risk is particularly high among patients with type 2 diabetes mellitus (T2DM), who account for approximately one-third of ACS cases [2, 3]. Because of the low acceptability of coronary artery bypass grafting (CABG) in Chinese patients with coronary artery disease (CAD), percutaneous coronary intervention (PCI) is currently the most common revascularization strategy, even in patients with diabetes who are more likely to develop multi-vessel CAD. However, PCI was demonstrated to be associated with a higher rate of short- and long-term adverse CV outcomes relative to CABG in diabetic patients with ACS and multi-vessel CAD [4]. Therefore, early identification of diabetic patients with ACS undergoing PCI who have a high residual risk is crucial for better clinical management to reduce future CV events.

Since 1988, insulin resistance (IR) has been established as a crucial mediator of metabolic disorders, T2DM, and atherosclerotic CV disease (CVD) [5–8]. Although the hyperinsulinaemic–euglycaemic clamp is the “gold standard” test for the measurement of IR [9], it is not commonly used in clinical settings due to the complexity of the testing process. IR is significantly associated with the chronic increase in plasma glucose and triglycerides [7]. Accordingly, it was speculated that the combination of plasma glucose and triglycerides could predict IR. Indeed, the product of fasting plasma glucose (FPG) and triglycerides, namely, the fasting triglyceride glucose (TyG) index, has been demonstrated to be significantly correlated with IR measured by the hyperinsulinaemic–euglycaemic clamp test and even perform better than homeostasis model assessment of IR (HOMA-IR) in non-diabetic as well as diabetic patients [10, 11].

Numerous clinical studies have indicated that the TyG index is associated with CVD morbidity and mortality in the general population and many patient cohorts including both non-diabetic and diabetic patients [12–20]; however, no previous study has exclusively investigated the prognostic usefulness of the TyG index for CV events after PCI in patients with T2DM and ACS. Therefore, we examined the relationship between the baseline TyG index and adverse CV outcomes in patients with T2DM and ACS who underwent PCI.

## Methods

### Study population

The present study is a retrospective analysis derived from a single-centre prospective observational study (ChiCTR1800017417) that aimed to explore the prognostic value of the Logistic Clinical SYNTAX Score and novel risk factors for major adverse CV events in ACS patients undergoing PCI. This prospective observational study was approved by the institutional review board of Beijing Anzhen Hospital, Capital Medical University, and all patients gave their written informed consent before study inclusion. Moreover, this prospective observational study included a prespecified subgroup of patients with diabetes, and all eligible patients were consecutively and prospectively enrolled in a customized database.

A total of 826 patients with T2DM who underwent coronary angiography for ACS and were treated with primary or elective PCI in our CV centre from June 2016 to November 2017 were consecutively and prospectively enrolled in a customized database. The exclusion criteria of this study included patients with prior CABG, cardiogenic shock, left ventricular ejection fraction (LVEF) < 30%, renal dysfunction with creatinine clearance (CrCl) < 15 mL/min or chronic dialysis, extreme body mass index (BMI > 45 kg/m^2^), suspected familial hypertriglyceridaemia [plasma triglycerides ≥ 500 mg/dL (5.65 mmol/L) or more than one first-degree relative with triglycerides ≥ 500 mg/dL]. Three patients were also excluded because of missing follow-up data despite at least 4 separate attempts to contact them. Ultimately, 776 patients were anonymously and retrospectively included in the final analysis. This study was performed in accordance with the Helsinki Declaration of Human Rights and approved by the institutional review board of Beijing Anzhen Hospital, Capital Medical University. Given the retrospective nature of this study, the requirement for informed consent was waived.

### Measurements

Data on demographics, personal medical history, and medication history were collected using a standard questionnaire. The blood pressure on admission was recorded. The concentrations of plasma triglycerides, total cholesterol (TC), high-density lipoprotein cholesterol (HDL-C), and glucose in the first fasting blood samples during the stay in the hospital, which were obtained after 12 h of fasting, were determined at the central laboratory of Beijing Anzhen Hospital. The low-density lipoprotein cholesterol (LDL-C) level was calculated using the Friedewald equation. The TyG index was retrospectively calculated using ln (fasting triglycerides [mg/dL] × fasting glucose [mg/dL]/2). Blood pressure was measured three times on different days, and readings ≥ 140/90 mmHg and antihypertensive medication use were considered criteria for hypertension. The symptoms of diabetes and a casual plasma glucose ≥ 200 mg/dL, FPG ≥ 126 mg/dL, 2-h plasma glucose concentration ≥ 200 mg/dL from a 75-g oral glucose tolerance test, and/or antidiabetic medication use were considered criteria for diabetes. Fasting TC > 200 mg/dL, LDL-C > 130 mg/dL, triglycerides > 150 mg/dL, HDL-C < 40 mg/dL, and/or chronic use of lipid-lowering drugs were considered criteria for dyslipidaemia. CrCl < 60 mL/min was considered the criterion for chronic kidney disease (CKD); CrCl was calculated using the Cockcroft and Gault formula [21]. Patients with previous ischaemic stroke or transient ischaemic attack were defined as having cerebrovascular accident (CVA). Patients with vascular diseases related to the aorta and arteries other than coronaries accompanied by exercise-related intermittent claudication, revascularization surgery, reduced or absent pulsation, angiographic stenosis of more than 50% or combinations of these characteristics were identified as having peripheral artery disease (PAD). Patients with signs/symptoms of congestive heart failure (CHF), current treatment for CHF, or objective evidence of reduced ejection fraction (LVEF < 40%) were considered to have cardiac failure.

### Follow-up details

All patients were followed up at 1 month and every 6 months after hospital discharge. The information regarding adverse events was obtained through telephone contact with the patients or their family members by trained personnel blinded to the baseline characteristics and ascertained from a careful review of corresponding medical records. The primary endpoint of the present study was the composite of overall death, non-fatal stroke, non-fatal myocardial infarction (MI), or unplanned repeat revascularization. Stroke was defined as ischaemic cerebral infarction with evidence of neurological dysfunction requiring hospitalization with clinically documented lesions on brain computed tomography or magnetic resonance imaging. MI was defined as an elevated level of cardiac troponin or creatine kinase (MB) greater than the upper limit of the normal range with either ischaemic symptoms or electrocardiographic changes implicating ischaemia. The presence of new pathological Q waves in ≥ 2 contiguous electrocardiogram leads was also diagnosed as MI. Within 1 week after the index PCI, only Q-wave MI was defined as MI. Unplanned repeat revascularization was defined as any non-staged revascularization after the index PCI. Staged revascularization was defined as scheduled revascularization within 90 days after the index PCI without treatment of a coronary artery territory that had been treated during the index PCI or a revascularization status of emergency, urgency, or salvage. The most severe endpoint event was selected for primary endpoint analysis if > 1 event occurred during follow-up (death > stroke > MI > revascularization). If more than one stroke or MI or revascularization occurred, the first stroke or MI or revascularization was selected. The follow-up period of the present study lasted until November 2019.

### Statistical analyses

Continuous variables were presented as the mean ± standard deviation if consistent with a normal distribution, otherwise as the median and interquartile range (IQR). Categorical variables were expressed as numbers and percentages. All patients were stratified into three groups (T1 [TyG index < 8.80], T2 [8.80 ≤ TyG index < 9.28], and T3 [TyG index ≥ 9.29]) in accordance with tertiles of the TyG index. The Chi squared test or Fisher’s exact test was used to analyse differences in categorical variables between groups. ANOVA or the Kruskal–Wallis H test was applied to analyse differences in continuous variables between groups. Kaplan–Meier methods were used to derive the event rates at follow-up and to plot time-to-event curves. Differences among Kaplan–Meier estimates of the three groups were evaluated with the log-rank test. We conducted a Cox proportional hazards regression analysis to estimate the hazard ratios (HRs) and their 95% confidence intervals (CIs) of developing the primary endpoint. The TyG index was analysed in two ways: (1) as a categorical variable; and (2) as a continuous variable. Predictors of the incidence of the primary endpoint identified through univariate analysis were also tested in a multivariate analysis. In the multivariate model, the following confounders were chosen because of their clinical importance and statistical significance in the univariate analysis: age (continuous), BMI (continuous), diastolic blood pressure (continuous), HDL-C (continuous), glycosylated haemoglobin (continuous), sex, current smoking, daily drinking, presence of PAD, CKD, cardiac failure, previous MI, past PCI, use of insulin and/or oral antidiabetic agents at discharge, CAD severity, presence of lesions > 20 mm long, use of drug-coated balloon, and complete revascularization. Interaction was tested with a likelihood ratio test, and the proportional hazard assumption was tested by demonstrating no importance of variables multiplied by time as time-dependent variables. Statistical analyses were performed using SPSS version 24.0 software (IBM Corporation, Chicago, IL), and *P* < 0.05 was considered statistically significant.

## Results

The mean age of the 776 patients at baseline was 61 ± 10 years, and 72.2% of patients were men (n = 560). The median follow-up duration was 30 months (IQR, 24–36 months), and during the follow-up period, 188 patients (24.2%) had at least 1 primary endpoint event, which was recorded in 40 (15.6%) patients from the T1 group, 66 (25.4%) from the T2 group, and 82 (31.7%) from the T3 group. In the 188 patients who had at least 1 primary endpoint event, there were 16 deaths (15 deaths from CV causes and 1 death from non-CV causes), 16 cases of non-fatal strokes, 21 cases of non-fatal MI, and 156 cases of unplanned revascularization. Of these, 19 (2.5%) patients suffered two, 2 (0.3%) patients suffered three, and 2 (0.3%) patients suffered four primary endpoint events. The respective incidences of overall death, CV death, non-CV death, non-fatal stroke, non-fatal MI and unplanned repeat revascularization among the TyG index tertiles are shown in Additional file 1: Table S1.

The baseline clinical and laboratory characteristics of the study patients stratified by the primary endpoint are summarized in Table 1. Compared with those without an endpoint event, patients with an endpoint event had higher levels of TyG index. Patients with an endpoint event also showed higher proportions of PAD, cardiac failure, previous MI, and past PCI, elevated concentrations of triglycerides and FPG but lower levels of HDL-C, DBP, and LVEF. Pre-hospital, periprocedural and discharge medications, angiographic findings, and procedural results of the study patients stratified by the primary endpoint are shown in Table 2. Pre-hospital, periprocedural and discharge medications, except for aspirin and insulin, did not differ between patients with and without an endpoint event. Compared with those without an endpoint event, patients with an endpoint event had higher rates of left-main/three-vessel disease, restenotic lesions, and lesions > 20 mm long. As regards procedural results, there were significant differences in the rates of drug-coated balloon use and complete revascularization between patients with and without an endpoint event.

**Table 1:** Baseline clinical and laboratory characteristics of the study patients stratified by the primary endpoint

Variable	No such events n = 588	Primary endpoint n = 188	*P* value
Demographics			
Age (years)	61 ± 10	62 ± 10	0.498
Male sex, n (%)	426 (72.4)	134 (71.3)	0.755
BMI (kg/m^2^)	26.2 ± 3.5	25.9 ± 3.1	0.298
Medical measurements (on admission)			
SBP (mm Hg)	132 ± 18	131 ± 15	0.339
DBP (mm Hg)	76 ± 11	73 ± 10	0.001
Risk factors			
Cigarette smoking			
Current smokers, n (%)	238 (40.5)	72 (38.3)	0.596
Former smokers, n (%)	89 (15.1)	35 (18.6)	0.257
Never smokers, n (%)	261 (44.4)	81 (43.1)	0.754
Alcohol intake			
Daily drinkers, n (%)	56 (9.5)	18 (9.6)	0.984
Family history of CHD, n (%)	172 (29.3)	66 (35.1)	0.130
Hypertension, n (%)	403 (68.5)	129 (68.6)	0.984
Dyslipidaemia, n (%)	483 (82.1)	163 (86.7)	0.145
Previous MI, n (%)	112 (19.0)	54 (28.7)	0.005
Past PCI, n (%)	120 (20.4)	66 (35.1)	< 0.001
Previous CVA, n (%)	37 (6.3)	11 (5.9)	0.827
PAD, n (%)	62 (10.5)	54 (28.7)	< 0.001
CKD, n (%)	40 (6.8)	20 (10.6)	0.087
Cardiac failure, n (%)	34 (5.8)	28 (14.9)	< 0.001
LVEF (%)	65 (60-68)	63 (58-68)	0.016
Clinical presentation			
UA, n (%)	457 (77.7)	153 (81.4)	0.287
NSTEMI, n (%)	82 (13.9)	18 (9.6)	0.119
STEMI, n (%)	49 (8.3)	17 (9.0)	0.762
Laboratory measurements (fasting state)			
TC (mg/dL)	157.6 ± 42.5	156.0 ± 37.4	0.638
LDL-C (mg/dL)	93.8 ± 34.9	91.6 ± 29.5	0.445
HDL-C (mg/dL)	39.8 ± 9.1	37.9 ± 7.9	0.008
Triglycerides (mg/dL)	128.4 (92.1–176.2)	145.2 (97.4–201.9)	0.039
FPG (mg/dL)	123.8 (109.9–147.2)	138.5 (114.2–169.4)	< 0.001
Glycosylated haemoglobin (%)	7.2 (6.6–8.1)	7.3 (6.8–8.2)	0.140
TyG index	9.02 ± 0.57	9.21 ± 0.53	< 0.001
TyG index tertiles			< 0.001
T1, n (%)	217 (36.9)	40 (21.3)	–
T2, n (%)	194 (33.0)	66 (35.1)	–
T3, n (%)	177 (30.1)	82 (43.6)	–

*BMI* body mass index, *SBP* systolic blood pressure, *DBP* diastolic blood pressure, *CHD* coronary heart disease, *MI* myocardial infarction, *PCI* percutaneous coronary intervention, *CVA* cerebrovascular accident, *PAD* peripheral artery disease, *CKD* chronic kidney disease, *UA* unstable angina, *NSTEMI* non ST-segment elevation myocardial infarction, *STEMI* ST-segment elevation myocardial infarction, *TC* total cholesterol, *LDL-C* low-density lipoprotein-cholesterol, *HDL-C* high-density lipoprotein-cholesterol, *FPG* fasting plasma glucose, *TyG* triglyceride glucose

**Table 2:** Pre-hospital, periprocedural and discharge medications, agiographic findings, and procedural results of the study patients stratified by the primary endpoint

Variable	No such events n = 588	Primary endpoint n = 188	*P* value
Medications before admission			
Aspirin, n (%)	432 (73.5)	146 (77.7)	0.251
P2Y12 inhibitors, n (%)	231 (39.3)	79 (42.0)	0.505
Lipid-lowering drugs, n (%)	430 (73.1)	144 (76.6)	0.346
ACEI/ARBs, n (%)	180 (30.6)	74 (39.4)	0.026
β-blockers, n (%)	227 (38.6)	71 (37.8)	0.837
Insulin, n (%)	209 (35.5)	73 (38.8)	0.415
Oral antidiabetic agents, n (%)	284 (48.3)	92 (48.9)	0.879
Metformin, n (%)	144 (24.5)	44 (23.4)	0.762
Alpha-glucosidase inhibitors, n (%)	123 (20.9)	31 (16.5)	0.185
Sulfonylurea, n (%)	135 (23.0)	43 (22.9)	0.980
Dipeptidyl peptidase 4 inhibitors, n (%)	8 (1.4)	4 (2.1)	0.687
Any antidiabetic treatment, n (%)	419 (71.3)	143 (76.1)	0.199
Periprocedural medications			
Aspirin, n (%)	588 (100.0)	182 (96.8)	< 0.001
P2Y12 inhibitors, n (%)	588 (100.0)	188 (100.0)	–
Unfractionated heparin, n (%)	482 (82.0)	156 (83.0)	0.754
Bivalirudin, n (%)	77 (13.1)	23 (12.2)	0.759
GP IIb/IIIa receptor antagonist, n (%)	100 (17.0)	42 (22.3)	0.100
Medications at discharge			
Aspirin, n (%)	588 (100.0)	182 (96.8)	< 0.001
P2Y12 inhibitors, n (%)	588 (100.0)	188 (100.0)	–
Lipid-lowering drugs, n (%)	588 (100.0)	188 (100.0)	–
ACEI/ARBs, n (%)	283 (48.1)	99 (52.7)	0.279
β-blockers, n (%)	432 (73.5)	130 (69.1)	0.249
Insulin, n (%)	188 (32.0)	76 (40.4)	0.033
Oral antidiabetic agents, n (%)	318 (54.1)	100 (53.2)	0.831
Metformin, n (%)	90 (15.3)	32 (17.0)	0.574
Alpha-glucosidase inhibitors, n (%)	214 (36.4)	64 (34.0)	0.558
Sulfonylurea, n (%)	146 (24.8)	42 (22.3)	0.488
Dipeptidyl peptidase 4 inhibitors, n (%)	8 (1.4)	4 (1.2)	0.687
Any antidiabetic treatment, n (%)	408 (69.4)	150 (79.8)	0.006
Angiographic findings			
One-vessel disease, n (%)	68 (11.6)	6 (3.2)	0.001
Two-vessel disease, n (%)	164 (27.9)	30 (16.0)	0.001
LM/three-vessel disease, n (%)	356 (60.5)	152 (80.9)	< 0.001
Proximal LAD stenosis, n (%)	291 (49.5)	103 (54.8)	0.206
Restenotic lesions, n (%)	61 (10.4)	49 (26.1)	< 0.001
Chronic total occlusions, n (%)	136 (23.1)	40 (21.3)	0.597
Trifurcation or bifurcation lesions, n (%)	450 (76.5)	148 (78.7)	0.534
Heavy calcification lesions, n (%)	193 (32.8)	65 (34.6)	0.657
Lesions > 20 mm long, n (%)	312 (53.1)	134 (71.3)	< 0.001
Procedural results			
Target vessel territory			
LM, n (%)	30 (5.1)	12 (6.4)	0.499
LAD, n (%)	291 (49.5)	91 (48.4)	0.796
LCX, n (%)	168 (28.6)	52 (27.7)	0.809
RCA, n (%)	231 (39.3)	75 (39.9)	0.882
DES use, n (%)	492 (83.7)	154 (81.9)	0.574
BRS use, n (%)	27 (4.6)	5 (2.7)	0.246
DCB use, n (%)	32 (5.4)	22 (11.7)	0.003
Complete revascularization, n (%)	376 (63.9)	80 (42.6)	< 0.001

*ACEI* angiotensin converting enzyme inhibitor, *ARB* angiotensin II receptor blocker, *LM* left-main artery, *LAD* left anterior descending artery, *LCX* left circumflex artery, *RCA* right coronary artery, *DES* drug-eluting stent, *BRS* bioresorbable scaffold, *DCB* drug-coated balloon

The baseline clinical and laboratory characteristics of the study patients according to the TyG index tertiles are presented in Table 3. Patients with a high TyG index were more likely to be men. BMI, TC level, LDL-C level, FPG level, triglyceride level and glycosylated haemoglobin level increased, whereas age and HDL-C level decreased in proportion to the TyG index tertiles. The proportion of current smokers and daily drinkers significantly increased with an increase in the TyG index. Pre-hospital, periprocedural and discharge medications, angiographic findings, and procedural results of the study patients according to the TyG index tertiles are listed in Table 4. Medications before admission and periprocedural medications were not different among the different TyG index groups. Medications at discharge, except for oral antidiabetic agents (mainly driven by the difference in α-glucosidase inhibitors), were similar across the groups. Among the patients within the top TyG index tertile, a significantly higher proportion had left-main coronary artery intervention. The rate of drug-eluting stent use was significantly lower in the patients within the top TyG index tertile.

**Table 3:** Baseline clinical and laboratory characteristics of the study patients according to the TyG index tertiles

Variable	T1 n = 257	T2 n = 260	T3 n = 259	*P* value
Demographics				
Age (years)	63 ± 9	62 ± 10	59 ± 11	< 0.001
Male sex, n (%)	173 (67.3)	201 (77.3)	186 (71.8)	0.040
BMI (kg/m^2^)	25.5 ± 3.4	26.1 ± 3.0	26.8 ± 3.8	< 0.001
Medical measurements (on admission)				
SBP (mm Hg)	132 ± 20	134 ± 18	130 ± 15	0.072
DBP (mm Hg)	74 ± 12	76 ± 10	76 ± 11	0.215
Risk factors				
Cigarette smoking				
Current smokers, n (%)	90 (35.0)	102 (39.2)	118 (45.6)	0.048
Former smokers, n (%)	41 (16.0)	51 (19.6)	32 (12.4)	0.078
Never smokers, n (%)	126 (49.0)	107 (41.2)	109 (42.1)	0.144
Alcohol intake				
Daily drinkers, n (%)	14 (5.4)	28 (10.8)	32 (12.4)	0.020
Family history of CHD, n (%)	69 (26.8)	87 (33.5)	82 (31.7)	0.242
Hypertension, n (%)	183 (71.2)	175 (67.3)	174 (67.2)	0.535
Dyslipidaemia, n (%)	163 (63.4)	234 (90.0)	249 (96.1)	< 0.001
Previous MI, n (%)	55 (21.4)	55 (21.2)	56 (21.6)	0.992
Past PCI, n (%)	64 (24.9)	68 (26.2)	54 (20.8)	0.335
Previous CVA, n (%)	18 (7.0)	16 (6.2)	14 (5.4)	0.753
PAD, n (%)	32 (12.5)	38 (14.6)	46 (17.8)	0.235
CKD, n (%)	20 (7.8)	22 (8.5)	18 (6.9)	0.812
Cardiac failure, n (%)	26 (10.1)	20 (7.7)	16 (6.2)	0.250
LVEF (%)	63 (60–67)	65 (60–68)	65 (60–68)	0.077
Clinical presentation				
UA, n (%)	211 (82.1)	194 (74.6)	205 (79.2)	0.112
NSTEMI, n (%)	26 (10.1)	42 (16.2)	32 (12.4)	0.117
STEMI, n (%)	20 (7.8)	24 (9.2)	22 (8.5)	0.840
Laboratory measurements (fasting state)				
TC (mg/dL)	141.2 ± 36.6	156.1 ± 38.2	174.1 ± 42.4	< 0.001
LDL-C (mg/dL)	82.3 ± 33.3	95.2 ± 32.0	102.2 ± 32.7	< 0.001
HDL-C (mg/dL)	42.2 ± 10.0	38.4 ± 7.7	37.4 ± 7.9	< 0.001
Triglycerides (mg/dL)	85.0 (70.8–100.1)	135.5 (113.3–157.6)	214.3 (169.1–267.4)	< 0.001
FPG (mg/dL)	116.0 (104.2–126.2)	124.2 (108.1–146.9)	147.6 (128.3–175.2)	< 0.001
Glycosylated haemoglobin (%)	6.9 (6.5–7.8)	7.3 (6.7–8.1)	7.6 (6.8–8.4)	< 0.001
TyG index	8.54 (8.32–8.68)	9.03 (8.93–9.14)	9.63 (9.44–9.89)	< 0.001

Abbreviations as in Table 1

**Table 4:** Pre-hospital, periprocedural and discharge medications, agiographic findings, and procedural results of the study patients according to the TyG index tertiles

Variable	T1 n = 257	T2 n = 260	T3 n = 259	*P* value
Medications before admission				
Aspirin, n (%)	190 (73.9)	197 (75.8)	191 (73.7)	0.843
P2Y12 inhibitors, n (%)	104 (40.5)	111 (42.7)	95 (36.7)	0.368
Lipid-lowering drugs, n (%)	196 (76.3)	193 (74.2)	185 (71.4)	0.454
ACEI/ARBs, n (%)	81 (31.5)	88 (33.8)	85 (32.8)	0.852
β-blockers, n (%)	108 (42.0)	104 (40.0)	86 (33.2)	0.097
Insulin, n (%)	88 (34.2)	108 (41.5)	86 (33.2)	0.099
Oral antidiabetic agents, n (%)	122 (47.5)	133 (51.2)	121 (46.7)	0.557
Metformin, n (%)	54 (21.0)	61 (23.5)	73 (28.2)	0.154
Alpha-glucosidase inhibitors, n (%)	50 (19.5)	58 (22.3)	46 (17.8)	0.422
Sulfonylurea, n (%)	50 (19.5)	72 (27.7)	56 (21.6)	0.069
Dipeptidyl peptidase 4 inhibitors, n (%)	6 (2.3)	4 (1.5)	2 (0.8)	0.313
Any antidiabetic treatment, n (%)	182 (70.8)	197 (75.8)	183 (70.7)	0.334
Periprocedural medications				
Aspirin, n (%)	255 (99.2)	260 (100.0)	255 (98.5)	0.113
P2Y12 inhibitors, n (%)	257 (100.0)	260 (100.0)	259 (100.0)	–
Unfractionated heparin, n (%)	213 (82.9)	213 (81.9)	212 (81.9)	0.944
Bivalirudin, n (%)	34 (13.2)	32 (12.3)	34 (13.1)	0.943
GP IIb/IIIa receptor antagonist, n (%)	40 (15.6)	50 (19.2)	52 (20.1)	0.371
Medications at discharge				
Aspirin, n (%)	255 (99.2)	260 (100.0)	255 (98.5)	0.113
P2Y12 inhibitors, n (%)	257 (100.0)	260 (100.0)	259 (100.0)	–
Lipid-lowering drugs, n (%)	257 (100.0)	260 (100.0)	259 (100.0)	–
ACEI/ARBs, n (%)	116 (45.1)	123 (47.3)	143 (55.2)	0.055
β-blockers, n (%)	189 (73.5)	190 (73.1)	183 (70.7)	0.733
Insulin, n (%)	86 (33.5)	94 (36.2)	84 (32.4)	0.653
Oral antidiabetic agents, n (%)	122 (47.5)	153 (58.8)	143 (55.2)	0.030
Metformin, n (%)	36 (14.0)	40 (15.4)	46 (17.8)	0.495
Alpha-glucosidase inhibitors, n (%)	76 (29.6)	103 (39.6)	99 (38.2)	0.036
Sulfonylurea, n (%)	56 (21.8)	72 (27.7)	60 (23.2)	0.260
Dipeptidyl peptidase 4 inhibitors, n (%)	4 (1.6)	6 (2.3)	2 (0.8)	0.365
Any antidiabetic treatment, n (%)	172 (66.9)	197 (75.8)	189 (73.0)	0.073
Angiographic findings				
One-vessel disease, n (%)	30 (11.7)	14 (5.4)	30 (11.6)	0.020
Two-vessel disease, n (%)	70 (27.2)	71 (27.3)	53 (20.5)	0.118
LM/three-vessel disease, n (%)	157 (61.1)	175 (67.3)	176 (68.0)	0.194
Proximal LAD stenosis, n (%)	117 (45.5)	143 (55.0)	134 (51.7)	0.091
Restenotic lesions, n (%)	38 (14.8)	34 (13.1)	38 (14.7)	0.823
Chronic total occlusions, n (%)	53 (20.6)	64 (24.6)	59 (22.8)	0.555
Trifurcation or bifurcation lesions, n (%)	199 (77.4)	205 (78.8)	194 (74.9)	0.557
Heavy calcification lesions, n (%)	90 (35.0)	96 (36.9)	72 (27.8)	0.067
Lesions > 20 mm long, n (%)	133 (51.8)	155 (59.6)	158 (61.0)	0.072
Procedural results				
Target vessel territory				
LM, n (%)	10 (3.9)	10 (3.8)	22 (8.5)	0.027
LAD, n (%)	132 (51.4)	120 (46.2)	130 (50.2)	0.461
LCX, n (%)	83 (32.3)	60 (23.1)	77 (29.7)	0.056
RCA, n (%)	93 (36.2)	122 (46.9)	91 (35.1)	0.010
DES use, n (%)	225 (87.5)	226 (86.9)	195 (75.3)	< 0.001
BRS use, n (%)	10 (3.9)	2 (0.8)	20 (7.7)	< 0.001
DCB use, n (%)	16 (6.2)	16 (6.2)	22 (8.5)	0.492
Complete revascularization, n (%)	165 (64.2)	148 (56.9)	143 (55.2)	0.089

Abbreviations as in Tables 1 and 2

**Fig. 1:**
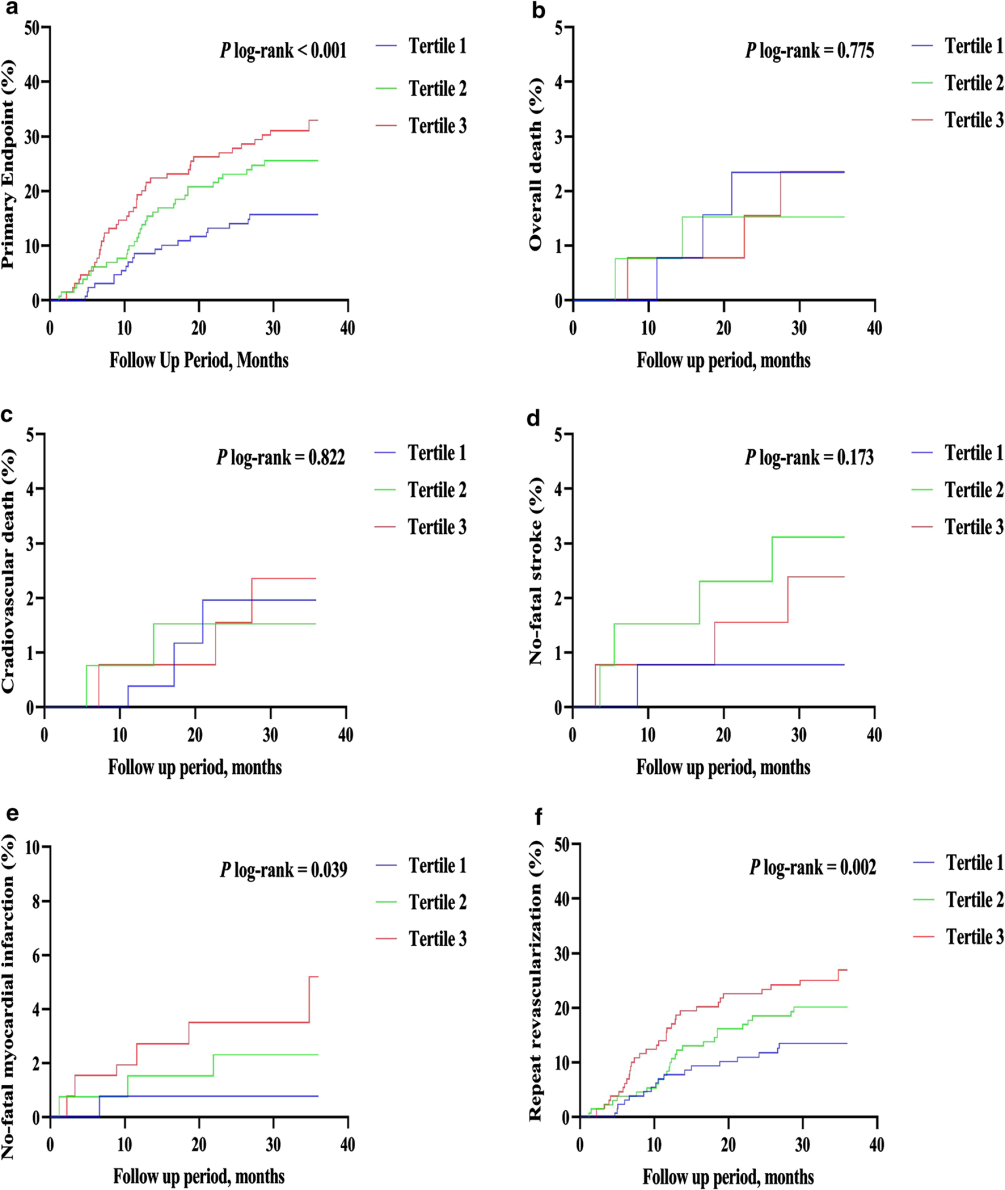
The TyG index and risk: Kaplan–Meier curves for the incidences of the primary endpoint (**a**), all-cause death (**b**), cardiovascular death (**c**), non-fatal stroke (**d**), non-fatal myocardial infarction (**e**), and unplanned repeat revascularization (**f**) among the 3 study groups based on the TyG index tertiles

Kaplan–Meier curves of the incidence of the primary endpoint and each component event of the primary endpoint for the TyG index tertiles are presented in Fig. 1. The incidence of the primary endpoint in the T3 group was significantly higher than that in the T1 group (*P* log-rank < 0.001). This difference was mainly driven by the increase in non-fatal MI (log-rank test, *P* = 0.039) and unplanned repeat revascularization (log-rank test, *P* = 0.002) across the TyG index tertiles. However, the incidence of overall death (log-rank test, *P* = 0.775), CV death (log-rank test, *P* = 0.822), and non-fatal stroke (log-rank test, *P* = 0.173) at follow-up were similar among the TyG index tertiles.

**Fig. 2:**
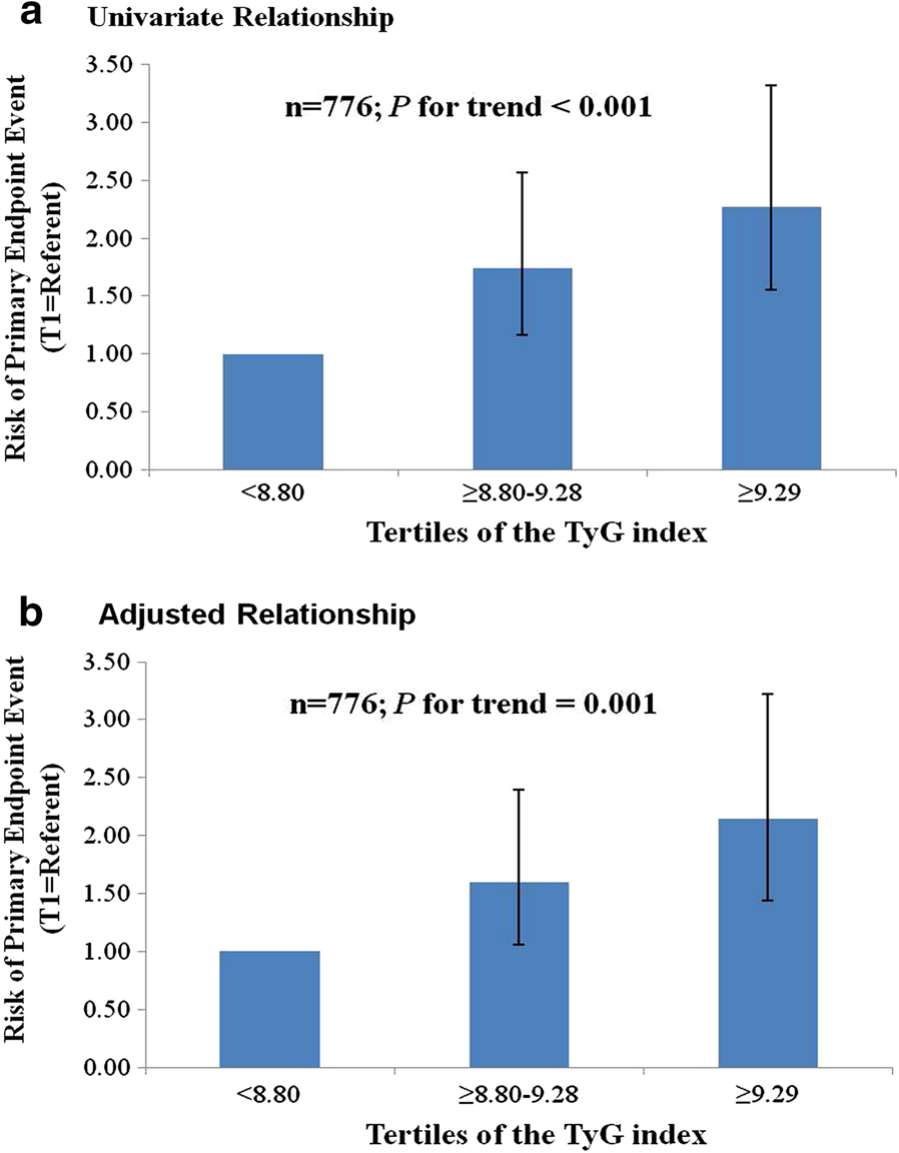
Risk of all-cause death, non-fatal stroke, non-fatal myocardial infarction, or unplanned repeat revascularization according to tertiles of the TyG index. Error bars indicate 95% confidence intervals. The first tertile is the reference. **a** Univariate relationship. **b** Relationship adjusted for age, body mass index, diastolic blood pressure, high-density lipoprotein cholesterol, glycosylated haemoglobin, sex, current smoking, daily drinking, presence of peripheral artery disease, chronic kidney disease, cardiac failure, previous myocardial infarction, past percutaneous coronary intervention, use of insulin and/or oral antidiabetic agents at discharge, coronary artery disease severity, presence of lesions > 20 mm long, use of drug-coated balloon, and complete revascularization

The TyG index at baseline was significantly related to the incidence of the primary endpoint. In univariate analysis, the TyG index as a continuous variable was associated with an HR of 1.64 (95% CI 1.15–1.97; *P* < 0.001). Adjustment for multiple confounders did not attenuate the relationship (HR 1.50, 95% CI 1.16–1.99; *P* = 0.003) (Additional file 2: Table S2). The incidence of the primary endpoint increased monotonically across the tertiles of the TyG index (*P* for trend ≤ 0.001) (Fig. 2). Taking T1 as the reference, multivariate analysis revealed that the TyG index for T2 and T3 increased the HRs for the incidence of the primary endpoint (T2: HR 1.60, 95% CI 1.06–2.40; T3: HR 2.15, 95% CI 1.44–3.22) (Table 5).

**Table 5:** Relationship between the incidence of the primary endpoint and the TyG index expressed as a categorical variable

Variables	Univariate analysis HR (95% CI)	*P* value	Multivariate analysis HR (95% CI)	*P*-value
TyG index tertiles				
T1	Reference		Reference	
T2	1.74 (1.17–2.57)	0.006	1.60 (1.06–2.40)	0.025
T3	2.27 (1.56–3.32)	< 0.001	2.15 (1.44–3.22)	< 0.001
Age	1.01 (0.99–1.02)	0.458	0.99 (0.97–1.004)	0.138
BMI	0.98 (0.94–1.02)	0.300	0.97 (0.92–1.01)	0.166
DBP	0.98 (0.96–0.99)	0.001	0.99 (0.97–1.00)	0.048
HDL-C	0.98 (0.96–1.00)	0.014	0.98 (0.96–1.00)	0.042
Glycosylated haemoglobin	1.05 (0.94–1.18)	0.387	0.93 (0.82–1.06)	0.307
Male sex	0.92 (0.67–1.26)	0.603	0.68 (0.44–1.04)	0.077
Current smoking	0.94 (0.70–1.27)	0.699	1.02 (0.71–1.46)	0.918
Daily drinking	1.01 (0.62–1.65)	0.957	1.30 (0.77–2.18)	0.330
Previous MI	1.52 (1.11–2.09)	0.009	0.88 (0.61–1.27)	0.500
Past PCI	1.79 (1.33–2.41)	< 0.001	1.72 (1.17–2.53)	0.006
PAD	2.82 (2.05–3.87)	< 0.001	2.22 (1.52–3.24)	< 0.001
CKD	1.70 (1.07–2.70)	0.025	1.46 (0.85–2.52)	0.172
Cardiac failure	2.27 (1.52–3.40)	< 0.001	1.59 (1.00–2.52)	0.049
Insulin at discharge	1.39 (1.04–1.87)	0.025	0.95 (0.67–1.36)	0.797
Metformin at discharge	1.12 (0.76–1.63)	0.568	1.08 (0.72–1.62)	0.707
Alpha-glucosidase inhibitors at discharge	0.89 (0.66–1.21)	0.457	0.76 (0.55–1.06)	0.104
Sulfonylurea at discharge	0.89 (0.64–1.26)	0.524	0.95 (0.65–1.38)	0.784
Dipeptidyl peptidase 4 inhibitors at discharge	1.34 (0.50–3.60)	0.566	1.24 (0.44–3.48)	0.685
CAD severity				
One-vessel disease	Reference		Reference	
Two-vessel disease	2.08 (0.87–5.01)	0.101	1.30 (0.52–3.24)	0.579
LM/three-vessel disease	4.32 (1.91–9.76)	< 0.001	2.16 (0.91–5.13)	0.079
Lesions > 20 mm long	2.07 (1.51–2.84)	< 0.001	1.59 (1.13–2.23)	0.008
DCB use	2.07 (1.33–3.23)	0.001	1.23 (0.73–2.08)	0.430
Complete revascularization	0.46 (0.35–0.62)	< 0.001	0.63 (0.46–0.86)	0.004

Abbreviations as in Tables 1 and 2

Relevant clinical variables like clinical presentation (unstable angina vs. acute MI), age (≤ 70 vs. > 70 years), sex (male vs. female), and BMI (< 28 vs. ≥ 28 kg/m^2^) were subject to post hoc subgroup analyses for the primary endpoint. When the analysis was stratified by clinical presentation, we found that a higher TyG index was significantly associated with an increased risk of the primary endpoint in patients with unstable angina (adjusted HR 1.52, 95% CI 1.13–2.05; *P* = 0.006), and the similar result occurred in patients with acute MI (adjusted HR 5.70, 95% CI 1.78–18.30; *P* = 0.003). When the analysis was stratified by age, we found that a higher TyG index was significantly associated with an increased risk of the primary endpoint in patients aged 70 years and younger (adjusted HR 1.77, 95% CI 1.30–2.42; *P* < 0.001); however, the similar result did not occur in patients aged over 70 years (adjusted HR 0.44, 95% CI 0.16–1.21; *P* = 0.110). When the analysis was stratified by sex, we found that a higher TyG index was significantly associated with an increased risk of the primary endpoint in male patients (adjusted HR 1.60, 95% CI 1.14–2.24; *P* = 0.007); however, the similar result did not occur in female patients (adjusted HR 1.11, 95% CI 0.66–1.88; *P* = 0.695). When the analysis was stratified by BMI, we found that a higher TyG index was significantly associated with an increased risk of the primary endpoint in patients with BMI < 28 kg/m^2^ (adjusted HR 1.43, 95% CI 1.06–1.93; *P* = 0.019), and the similar result occurred in patients with BMI ≥ 28 kg/m^2^ (adjusted HR 12.07, 95% CI 3.32–43.92; *P* < 0.001).

## Discussion

In the present study, we noticed a significant association between the TyG index and adverse CV outcomes in patients with T2DM and ACS who underwent PCI. Even after adjustment for as many potential confounding risk factors as possible, an independent association of the TyG index with adverse CV outcomes remained. To the best of our knowledge, this is the first study to investigate the prognostic value of the TyG index in diabetic patients with ACS undergoing PCI.

The TyG index was originally studied as a marker of identifying IR, with Pearson’s correlation coefficients of − 0.68 with M rates as measured by the hyperinsulinaemic-euglycaemic clamp test, which is the “gold standard” test in the diagnosis of IR [10], and 0.32 with the HOMA-IR, which is the most widely used method in current clinical practice for the estimation of IR [22]. Compared with several lipid ratios (TC/HDL-C, non-HDL-C/HDL-C, LDL-C/HDL-C, triglyceride/HDL-C, and apolipoprotein B/apolipoprotein A1), the visceral adiposity index, and the lipid accumulation product, the TyG index had a more significant association with HOMA-IR and showed a better discriminatory performance for HOMA-IR [23].

The TyG index, as a surrogate marker of IR, was demonstrated to be a useful predictor of metabolic syndrome and T2DM. Subsequently, a number of clinical studies were conducted to investigate the association of the TyG index with CVD morbidity and mortality in the general population and many patient cohorts, including both non-diabetic and diabetic patients. A population-based, prospective cohort study including 5014 apparently healthy subjects demonstrated that a higher TyG index was associated with an increased risk of incident CVD (CHD, PAD and CVA), independent of diabetic status [12]. Similarly, another population-based but retrospective cohort study including 6078 apparently healthy subjects over 60 years old also demonstrated that an elevated TyG index was significantly associated with an increased risk of developing CVD events, including both fatal and non-fatal CHD and CVA, independent of diabetic status [13]. Alizargar et al. showed that the TyG index was significantly correlated with the total amount of carotid plaque and the intima-media thicknesses of the internal, external, and common carotid arteries in hypertensive and normotensive community-dwelling individuals [14]. Alessandra et al. showed that the TyG index was positively associated with a higher prevalence of symptomatic CAD and could be used as a marker of atherosclerosis in patients with known CVD [15]. Moreover, Jin et al. showed that the TyG index was positively associated with future CV events, including all-cause death, non-fatal MI, stroke and post-discharge revascularization, suggesting that the TyG index may be a useful marker for predicting clinical outcomes in patients with stable CAD [16]. The findings of Mao et al. showed that the TyG index might be an independent predictor of CAD severity as evaluated by the SYNTAX Score and future CV events defined as the composite of cardiac death, non-fatal MMI, target vessel revascularization, CHF, and non-fatal stroke in non-ST-segment elevation ACS [17]. In addition, the findings of Luo et al. showed that a higher TyG index was associated with an increased risk of future CV events, including all-cause death, target vessel revascularization, non-fatal MI, unstable angina requiring hospitalization, CHF, stroke or transient cerebral ischaemia, in ST-segment elevation MI patients undergoing PCI [18]. Furthermore, the association between the TyG index and CV risk has also been specifically explored in patients with diabetes. A cross-sectional study including 888 asymptomatic subjects with T2DM but without a previous history of CHD showed that a higher TyG index was associated with an increased risk of significant coronary artery stenosis [19]. A nested case–control study including 1282 patients with T2DM and stable CAD showed that the TyG index was positively associated with future CV events, which were defined as all-cause death, non-fatal MI, stroke and post-discharge revascularization [20]. Additionally, a cohort study including 25,969 participants without previous diabetes or CVD indicated substantial similarities in the inflammatory profiles associated with diabetes and CVD [24]. Recently, a mediation analysis was performed to quantify the magnitude and relative contributions of several traditional or non-traditional CV risk factors in the pathway from T2DM to increased CV events (MI, stroke and vascular mortality) and demonstrated that the most important pathway contributing to CV events was the presence of IR assessed by the TyG index, followed by elevated triglycerides, the presence of microalbuminuria and reduced kidney function, whereas excess risk was not mediated through elevated systolic blood pressure or high LDL-C [25].

Although the mechanism underlying the association of the TyG index with adverse CV outcomes has not been elucidated, it may be linked to IR. In recent years, many studies have indicated the importance of IR not only in atherogenesis but also in advanced plaque progression by promoting apoptosis of macrophages, endothelial cells, and vascular smooth muscle cells [8, 26, 27]. Several metabolic changes caused by IR can induce the development of CVD. For example, IR can induce an imbalance in glucose metabolism that generates chronic hyperglycaemia, which in turn triggers oxidative stress and causes an inflammatory response that leads to vascular endothelial cell damage. IR can also alter lipid metabolism, which then leads to the development of dyslipidaemia and the well-known lipid triad: (1) elevated plasma triglycerides, (2) reduced plasma HDL-C, and (3) the appearance of small dense LDL-C particles. These metabolic changes contribute to atherosclerotic plaque formation [7]. Moreover, IR accompanied by hyperglycaemia and hypertriglyceridaemia has been demonstrated to be correlated with elevated plasminogen activator inhibitor-1 levels, leading to decreased fibrinolytic activity and increased thrombotic events [28, 29]. Furthermore, IR has been shown to be associated with structural and functional arterial wall injuries, such as endothelial dysfunction, impaired vasodilation, increased arterial stiffness, increased intima-media thickness, and increased coronary artery calcification, which are highly predictive of future CV events [30]. Notably, several studies confirmed that in the setting of acute MI, IR was associated with myocardial and microvascular injury. For example, Trifunovic et al. reported that IR was independently associated with poorer myocardial reperfusion assessed by the residual ST-segment elevation and impaired coronary microcirculatory function estimated by coronary flow reserve and potentially associated with larger final infarct size evaluated by the fixed perfusion defect on single-photon emission computed tomography myocardial perfusion imaging in non-diabetic patients with ST-segment elevation MI treated by primary PCI [31]. The poorer myocardial reperfusion, impaired coronary microcirculatory function and larger final infarct size after primary PCI were all related to the subsequent increase in short- and long-term adverse CV events. In fact, the association of IR with adverse CV outcomes has been proven in several clinical studies. A prospective Danish population-based study involving 2493 apparently healthy subjects reported that IR, assessed as HOMA-IR, was associated with incident CV events (CV death, non-fatal ischaemic heart disease, and non-fatal stroke) independent of metabolic syndrome based on both the International Diabetes Foundation and the National Cholesterol Education Program criteria [32]. A similar result was observed in a population of patients who had developed CVD. A multicentre prospective study including 2938 patients with pre-existing CAD revealed that IR, evaluated by HOMA-IR, was a good predictor for CV events (fatal and non-fatal MI and sudden death) [33]. Furthermore, aggressive treatment of diabetes and IR might significantly reduce the risk of CV events. Pioglitazone primarily stimulates peroxisome proliferator-activated receptor (PPAR)-gamma and partially activates PPAR-α, which ameliorates IR and improves glucose and lipid metabolism. Multiple studies have demonstrated that pioglitazone reduces future CV events in association with enhanced insulin sensitivity [8]. In a multicentre, randomized controlled trial including 5238 patients with T2DM who had evidence of macrovascular disease, pioglitazone was demonstrated to reduce the composite of all-cause mortality, non-fatal MI, and stroke [34]. Additionally, in another multicentre, randomized controlled trial involving 3876 patients without diabetes who had a recent history of ischaemic stroke or transient ischaemic attack and who had IR, the patients who received pioglitazone had a lower risk of fatal or non-fatal stroke or MI than those who received placebo [35]. Moreover, a recently published meta-analysis of randomized, controlled trials reported that pioglitazone significantly reduced the composite endpoints of non-fatal MI, non-fatal stroke and CV death in people with IR, pre-diabetes and diabetes mellitus [36].

The TyG index per se was reported to be positively associated with arterial stiffness as assessed by brachial-ankle or carotid-femoral pulse wave velocity, which have been demonstrated to predict the long-term risk of adverse CV events in patients with T2DM and ACS [37–39]. Of note, the TyG index includes triglycerides and glucose in its formula. Fasting triglycerides and glucose were shown to be associated with both long-term and short-term CV risk after ACS, independent of diabetic status [40, 41]. The aforementioned relationships might in part explain the potential association between the TyG index and adverse CV outcomes in diabetic patients with ACS. Moreover, we found that BMI and LDL-C levels were significantly higher in patients in the highest TyG index tertile than in those in other tertiles, whereas HDL-C levels were lower. Therefore, the association of the TyG index with adverse CV outcomes may be partially mediated by these traditional CV risk factors.

Our study also had several important limitations. First, this was a retrospective analysis derived from a single-centre prospective observational study, which could not definitively establish causality. Second, the baseline levels of FPG and triglycerides could be affected by the use of lipid-lowering drugs and antidiabetic agents before admission. However, there were no significant differences among the three groups on the basis of the TyG index tertiles with respect to the use of lipid-lowering drugs and antidiabetic agents before admission. Third, none of the study patients were treated with drugs specifically designed to lower triglycerides, such as fibrates, niacin, and omega-3 fatty acids, before admission and at discharge; therefore, our results may not be applicable to patients treated with such drugs. Fourth, whether the findings from the present study including only Chinese patients can be extrapolated to other ethnic groups will require further studies. Fifth, we did not compare the TyG index with HOMA-IR and the hyperinsulinaemic–euglycaemic clamp test. Finally, we did not record nutritional habits or energy intake. Although we did not adjust for these potential confounding variables, we used adjusted other variables, such as BMI or cholesterol levels which are indirectly related to nutritional habits or energy intake.

## Conclusions

The TyG index, which is easily measurable and applicable in clinical practice without the need for complicated techniques or formulas, was significantly associated with a higher risk of CV events in patients with T2DM and ACS who underwent PCI, and this relationship remained significant after adjustment for other confounders. Further prospective, large studies are required to confirm our findings.

## Supplementary information

**Additional file 1: Table S1.** Adverse CV events according to the TyG index tertiles during follow-up.

**Additional file 2: Table S2.** Relationship between the incidence of the primary endpoint and the TyG index expressed as a continuous variable.

## Data Availability

The datasets used during the current study are available from the corresponding author on reasonable request.

## Abbreviations

ACS: Acute coronary syndrome
BMI: Body mass index
CABG: Coronary artery bypass grafting
CAD: Coronary artery disease
CHF: Congestive heart failure
CI: Confidence interval
CKD: Chronic kidney disease
CrCl: Creatinine clearance
CV: Cardiovascular
CVA: Cerebrovascular accident
CVD: Cardiovascular disease
FPG: Fasting plasma glucose
HDL-C: High-density lipoprotein cholesterol
HOMA-IR: homeostasis model assessment of insulin resistance
HR: Hazard ratio
IQR: Interquartile range
IR: Insulin resistance
LDL-C: Low-density lipoprotein cholesterol
LVEF: Left ventricular ejection fraction
MI: Myocardial infarction
PAD: Peripheral artery disease
PCI: Percutaneous coronary intervention
PPAR: Peroxisome proliferator-activated receptor
T2DM: Type 2 diabetes mellitus
TC: Total cholesterol
TyG: Triglyceride glucose
