# Supplementary material for: Triglyceride glucose index for predicting cardiovascular outcomes after percutaneous coronary intervention in patients with type 2 diabetes mellitus and acute coronary syndrome
Source: Cardiovasc Diabetol. 2020 Mar 10;19:31. doi: 10.1186/s12933-020-01006-7 (PMC7063826; doi:10.1186/s12933-020-01006-7)
Supplement: Supplementary file 1 — Additional file 1: Table S1. Adverse CV events according to the TyG index tertiles during follow-up. [file 12933_2020_1006_MOESM1_ESM.docx]

Table S1. Adverse CV events according to the TyG index tertiles during follow-up

| CV events | T1  n =257 | T2  n =260 | T3  n =259 | *P* Value |
| --- | --- | --- | --- | --- |
| Primary endpoint, n (%) | 40 (15.6) | 66 (25.4) | 82 (31.7) | <0.001 |
| Death, n (%)  CV causes  Non-CV causes | 6 (2.3)  5 (1.9)  1 (0.4) | 4 (1.5)  4 (1.5)  0 (0) | 6 (2.3)  6 (2.3)  0 (0) | 0.767  0.811  0.331 |
| Non-fatal stroke, n (%) | 2 (0.8) | 8 (3.1) | 6 (2.3) | 0.173 |
| Non-fatal MI, n (%) | 2 (0.8) | 6 (2.3) | 11 (4.2) | 0.038 |
| Unplanned repeat revascularization, n (%) | 34 (13.2) | 52 (20.0) | 66 (25.5) | 0.002 |

The primary endpoint was defined as a composite of overall death, non-fatal stroke, non-fatal MI, and unplanned repeat revascularization.

TyG indicates triglyceride glucose; CV cardiovascular; MI, myocardial infarction.
