# Supplementary material for: Triglyceride glucose index for predicting cardiovascular outcomes after percutaneous coronary intervention in patients with type 2 diabetes mellitus and acute coronary syndrome
Source: Cardiovasc Diabetol. 2020 Mar 10;19:31. doi: 10.1186/s12933-020-01006-7 (PMC7063826; doi:10.1186/s12933-020-01006-7)
Supplement: Supplementary file 2 — Additional file 2: Table S2. Relationship between the incidence of the primary endpoint and the TyG index expressed as a continuous variable. [file 12933_2020_1006_MOESM2_ESM.docx]

Table S2. Relationship between the incidence of the primary endpoint and the TyG index expressed as a continuous variable.

| Variables | Univariate analysis  HR (95% CI) | *P*-value | Multivariate analysis  HR (95% CI) | *P*-value |
| --- | --- | --- | --- | --- |
| TyG index | 1.64 (1.29-2.09) | <0.001 | 1.50 (1.15-1.97) | 0.003 |
| Age | 1.01 (0.99-1.02) | 0.458 | 0.98 (0.97-1.01) | 0.154 |
| BMI | 0.98 (0.94-1.02) | 0.300 | 0.97 (0.92-1.02) | 0.173 |
| DBP | 0.98 (0.96-0.99) | 0.001 | 0.99 (0.97-1.00) | 0.057 |
| HDL-C | 0.98 (0.96-1.00) | 0.014 | 0.98 (0.96-1.00) | 0.083 |
| Glycosylated haemoglobin | 1.05 (0.94-1.18) | 0.387 | 0.95 (0.83-1.08) | 0.406 |
| Male sex | 0.92 (0.67-1.26) | 0.603 | 0.71 (0.46-1.08) | 0.110 |
| Current smoking | 0.94 (0.70-1.27) | 0.699 | 1.01 (0.70-1.45) | 0.969 |
| Daily drinking | 1.01 (0.62-1.65) | 0.957 | 1.34 (0.80-2.25) | 0.266 |
| Previous MI | 1.52 (1.11-2.09) | 0.009 | 0.91 (0.63-1.32) | 0.621 |
| Past PCI | 1.79 (1.33-2.41) | <0.001 | 1.70 (1.16-2.50) | 0.007 |
| PAD | 2.82 (2.05-3.87) | <0.001 | 2.17 (1.48-3.17) | <0.001 |
| CKD | 1.70 (1.07-2.70) | 0.025 | 1.46 (0.85-2.51) | 0.166 |
| Cardiac failure | 2.27 (1.52-3.40) | <0.001 | 1.53 (0.97-2.43) | 0.069 |
| Insulin at discharge | 1.39 (1.04-1.87) | 0.025 | 0.95 (0.67-1.35) | 0.760 |
| Metformin at discharge | 1.12 (0.76-1.63) | 0.568 | 1.08 (0.72-1.63) | 0.695 |
| Alpha-glucosidase inhibitors at discharge | 0.89 (0.66-1.21) | 0.457 | 0.78 (0.57-1.08) | 0.141 |
| Sulfonylurea at discharge | 0.89 (0.64-1.26) | 0.524 | 0.94 (0.64-1.37) | 0.745 |
| Dipeptidyl peptidase 4 inhibitors at discharge | 1.34 (0.50-3.60) | 0.566 | 1.21 (0.43-3.40) | 0.712 |
| CAD severity |  |  |  |  |
| One-vessel disease | Reference |  | Reference |  |
| Two-vessel disease | 2.08 (0.87-5.01) | 0.101 | 1.22 (0.49-3.04) | 0.673 |
| LM/three vessel disease | 4.32 (1.91-9.76) | <0.001 | 2.17 (0.92-5.12) | 0.078 |
| Lesions >20 mm long | 2.07 (1.51-2.84) | <0.001 | 1.59 (1.13-2.22) | 0.007 |
| DCB use | 2.07 (1.33-3.23) | 0.001 | 1.23 (0.73-2.07) | 0.433 |
| Complete revascularization | 0.46 (0.35-0.62) | <0.001 | 0.62 (0.45-0.85) | 0.003 |

Abbreviations as in Tables 1 and 2.
